# Distribution of drug-resistant genes in alveolar lavage fluid from patients with psittacosis and traceability analysis of causative organisms

**DOI:** 10.3389/fmicb.2023.1182604

**Published:** 2023-06-22

**Authors:** Huiqun Lu, Jing Yuan, Zeming Wu, Lingwei Wang, Shuang Wu, Qiongcheng Chen, Zhen Zhang, Zhigao Chen, Xuan Zou, Qinghua Hu, Tiejian Feng, Jianhua Lu, Liyin Ji, Shuxiang Qiu, Shiqin Xu, Min Jiang, Yinghui Li, Bo Peng, Qinqin Bai, Rui Cai, Yijie Geng, Xiaolu Shi

**Affiliations:** ^1^Department of Public Health Laboratory Sciences, School of Public Health, University of South China, Hengyang, China; ^2^Shenzhen Third People’s Hospital, Second Affiliated Hospital of Southern University of Science and Technology, Shenzhen, China; ^3^Shenzhen Hospital of Guangzhou University of Chinese Medicine (Futian), Shenzhen, China; ^4^Shenzhen People’s Hospital, Shenzhen, China; ^5^Shenzhen Center for Disease Control and Prevention, Shenzhen, China

**Keywords:** psittacosis, metagenomic next-generation sequencing, genotyping, host traceability, antibiotics

## Abstract

**Background:**

*Chlamydia psittaci* is a small bacterium often found in birds, including poultry, and domesticated mammals, which causes psittacosis (or parrot fever) in humans. Different strains of *C. psittaci* respond variably to antibiotics, suggesting a possible risk of antibiotic resistance. In general, different genotypes of *C. psittaci* have relatively stable hosts and different pathogenicity.

**Methods:**

Macrogenomic sequencing was performed using nucleic acids extracted from psittacosis patients’ alveolar lavage fluid samples and analyzed for genetic variability and antibiotic resistance genes. Nucleic acid amplification sequences specific to the core coding region of the *C. psittaci ompA* gene were used, and a phylogenetic tree was constructed with *C. psittaci* genotypic sequences from other sources, including Chinese published sources. The *C. psittaci* found in each patient were genotyped by comparing *ompA* gene sequences. In addition, to better illustrate the relationship between genotype and host of *C. psittaci*, 60 bird fecal samples were collected from bird-selling stores for screening and *C. psittaci* typing.

**Results:**

Macrogenomic sequence alignment revealed the presence of resistance genes in varying abundance in samples from all three patients, including *C. psittaci* resistance gene sequences from two patients that matched those previously published on NCBI. Based on *ompA* genotyping, two patients were infected with *C. psittaci* genotype A and one patient was infected with genotype B. All five *C. psittaci*-positive samples obtained from bird-selling stores were genotype A. Both genotypes are reported to be infectious to humans. The host origin of the samples and the previously reported main sources of each genotype suggested that all but one of the *C. psittaci* genotype A in this study were derived from parrots, while genotype B was probably derived from chickens.

**Conclusion:**

The presence of bacterial resistance genes in psittacosis patients may affect the efficacy of clinical antibiotic therapy. Focusing on the developmental progression of bacterial resistance genes and differences in the therapeutic efficacy may facilitate effective treatment of clinical bacterial infections. Pathogenicity genotypes (e.g., genotype A and genotype B) are not limited to one animal host, suggesting that monitoring the development and changes of *C. psittaci* may help prevent transmission to humans.

## 1. Introduction

Parrot fever, also known as psittacosis, is a human-animal disease caused by *Chlamydia psittaci*, which is hosted primarily by birds and livestock. Based on *ompA* genotyping, *C. psittaci* is currently divided into 15 main genotypes, seven of which were originally isolated from birds and are reported to infect humans: A (parrots), B (pigeons), C (ducks and geese), D (turkeys), E (multiple hosts), F (parrots and turkeys), and E/B (parrots and ducks) (Sachse et al., 2009). Additionally, M56 and WC are mainly found in mammals; Mat116, 6N, 1V, YP84, CPX0308, R54 are provisional genotypes originally isolated from birds (Supplementary Material 1; Pang et al., 2021).

Patients with parrot fever often present with non-specific clinical signs, such as high fever, headache, muscle aches, and atypical pneumonia, which are difficult to distinguish from diseases caused by *Legionella*, *Cryptococcus*, *Mycoplasma pneumoniae*, and influenza viruses (Zhang et al., 2021; Qu et al., 2022). Failure to diagnose psittacosis in a timely manner or the provision of inappropriate antibiotic treatment may lead to exacerbation of the patient’s condition, the development of severe atypical pneumonia, and even death (Beeckman and Vanrompay, 2009; Stokes et al., 2021). Such non-specific symptoms mean psittacosis is difficult to recognize and initial treatment is usually based on empirical treatment by physicians or by referring to the treatment protocol for community-acquired infections (Chen et al., 2020). The most commonly used antibiotics in the treatment protocol for community-acquired bacterial infections are beta-lactamases, such as penicillin (29%), amoxicillin (16%), and piperacillin (8%), followed by third-generation cephalosporins (mainly ceftriaxone, 16%), and fluoroquinolones (14%) (Mandell et al., 2007; Cillóniz et al., 2019). However, mice infected with *C. psittaci* treated with amoxicillin at an inoculation dose of 20 mg/kg coexisted with the pathogen in a non-infectious, viable state, with the antibiotic failing to completely kill the *C. psittaci*, suggesting that the treatment of *Chlamydia* with this class of drugs is somewhat restricted (Chen et al., 2021). Antibiotics commonly used in the clinical treatment of *Chlamydia spp*. include tetracyclines, macrolides, quinolones, rifampicin and lincosamides (Benamri et al., 2021; Yang et al., 2022), with tetracyclines the most commonly used, followed by quinolones (Bommana and Polkinghorne, 2019; Xiao et al., 2021). *In vitro* studies with the quinolone moxifloxacin have shown good antibacterial effects against *C. pneumoniae*, *C. trachomatis* and *C. psittaci*, which can each cause disease in humans (Donati et al., 1999).

The difficulty in psittacosis diagnosis using traditional methods has led to few cases being reported. In recent years, however, the development of molecular diagnostics and in-depth research, metagenomic next-generation sequencing (mNGS) has been applied to the screening of pathogens in disease cases difficult to diagnose, such as pneumonia of unknown origin (Zhang J. et al., 2022). The application of this technology has provided ample reference data for early diagnosis and treatment of clinical diseases, including a significant increase in the number of confirmed and reported cases of psittacosis.

We analyzed the association between antibiotic resistance genes and clinical treatment through mNGS data of alveolar lavage fluid from three psittacosis patients. Based on the contact history of patients, the genotype of *C. psittaci* and correlation with the source of the strain, we propose targeted implementation of effective preventive measures at the source to reduce human infections.

## 2. Materials and methods

### 2.1. The 3 patient cases

Case 1 (sample number Cps2), a 73-year-old male residing in Longgang District, Shenzhen, started to experience fever on May 13, 2022, with temperature up to 39.5°C and chills. Coughing began on May 14, without sputum; shortness of breath and diarrhea appeared on May 15, producing yellow paste-like stools. The patient was admitted to the hospital on the same day. After admission, the patient was injected with cefoperazone sodium and sulbactam sodium (cephalosporin antibiotic) for anti-infection, and was treated with gastric protection, blood pressure lowering, and low-flow oxygenation. One day later, the patient’s shortness of breath was still evident, and the index of infection was significantly elevated, so he was changed to high flow wetting therapy and treated with combined meropenem (carbapenem antibiotic) and doxycycline (tetracycline antibiotic). However, the patient still had recurrent fever with a maximum temperature of 39.0°C, cough, no improvement in shortness of breath, no significant coughing of sputum, decreased oxygenation index, oxygen saturation still maintained between 92 and 94%, and respiration at approximately 30–40 breaths per minute. On May 17, he was transferred to ICU and given moxifloxacin (quinolone antibiotic) combined with Tergesicin (penicillin antibiotic). The CT examination of the chest showed lamellar liquid density shadows in the pleural cavity bilaterally, large lamellar hyperdensity shadows in the lower lobe of both lungs and the upper lobe of the left lung, partly with air-containing bronchial shadows. There was also heart enlargement, abnormal density shadows in the pericardial cavity, and high-density shadows in the aortic travel area. Concurrently, yellow-green sputum was observed in the bronchial tubes of the lower lobe of the left lung on fibroneuroscopy, and subsequently sputum and blood specimens were retained and sent for sequence testing. On May 18, mNGS results of the patient’s sputum indicated *C. psittaci* infection. The patient was treated with moxifloxacin in combination with doxycycline, but the patient’s oxygenation index did not improve, and a repeat chest radiograph indicated that the left lung lesion continued to progress. The patient’s fever improved, the infection index gradually decreased, oxygenation improved, and the review chest X-ray indicated that the left lung infection absorption had improved. On May 20, treatment continued with extracorporeal artificial membrane lung, postoperative continuous heparin sodium anticoagulation moxifloxacin combined with doxycycline against *C. psittaci* infection, and vancomycin (glycopeptide) to prevent positive coccidioides infection. Function tests, however, indicated liver and kidney damage and so vancomycin was discontinued, resulting in liver and kidney function improvement. On May 24, the patient discontinued the extracorporeal lung treatment and continued ventilator-protected pulmonary ventilation. The next day, a repeat chest CT scan showed that the infection in the left lung, oxygenation index, and the shortness of breath had improved. Doxycycline was continued orally from May 30 to June 5, and the patient was discharged from the hospital on June 9. The patient keeps parrots at home, and the feeding of the parrots and the cleaning of the aviary are done by the patient. The patient’s parrot had mild diarrhea prior to the illness, subsequently died and was disposed of by the patient.

Case 2 (sample number Cps4), a 64-year-old female living in Luohu District, Shenzhen, China, began to have a persistent fever without obvious cause on June 24, 2022. Initially the patient had a temperature of approximately 38.5°C, which gradually increased to 40°C, which was accompanied by chills, severe headache, fatigue and muscle pain, and loss of appetite. On the third day, she first visited the hospital and was thought to have a viral infection. After treatment with ibuprofen and antiviral pellets, her body temperature temporarily decreased to normal then subsequently rose to 40°C. On June 29, she visited the hospital again and was found to have an elevated ultrasensitive C-reactive protein of 151.24 mg/L, a white blood cell count of 4.59 × 10^9^/L, a central granulocyte ratio of 77.8%, and a routine blood count of 3.79 × 10^9^/L, PCT: 1.98 ng/ml, ultrasensitive CRP 193.44 mg/L. A chest CT scan suggested the presence of a right lower lobe lung infection, and cefoperazone sulbactam (cephalosporins) and abirater were administered to treat the infection. On July 2, mNGS confirmed diagnosis of *C. psittaci* infection and the patient was given doxycycline and azithromycin (macrolides), which relieved the symptoms. According to the patient, she did not have direct contact with live birds or poultry, nor did she walk or stay in a park where birds were present. However, she may have passed through a poultry vending stand during her regular visits to the vegetable market.

Case 3 (sample number Cps5), a 50-year-old male, residing in Futian District, Shenzhen, presented to the hospital on July 19, 2022, with a severe cough, predominantly a dry cough at night, accompanied by fever with a peak of 38°C, itchy throat, occasional dizziness, feeling of drowsiness and general malaise. A chest CT scan revealed inflammation of the lower lobe of the left lung and ground glass nodules in both lungs. The patient had a small amount of effusion in the left side of the lung. The blood count was 16.60%, the lymphocyte count was 1.06 × 10^9^/L, the white blood cell count was 6.34 × 10^9^/L. The liver function was 79.20 U/L for aspartate aminotransferase and 114.76 U/L for glutamatergic aminotransferase; the renal function and the hepatobiliary, pancreatic, and splenic ultrasound did not show any significant abnormalities. During his stay in the hospital, he was conscious, mentally lethargic, with intermittent cough, dry cough, fever (peak 38.7°C), occasional chills, sweating, general malaise, itchy throat, occasional dizziness, and a feeling of drowsiness. After the patient was not significantly treated with levofloxacin hydrochloride (a quinolone antibiotic), the doctor changed the medication to two other types of cephalexin granules (a cephalosporin antibiotic) and doxycycline tablets, commonly used for lung infections, and the patient’s symptoms were significantly reduced. Fluorescent PCR testing, combined with mNGS, confirmed that the patient’s disease was *C. psittaci* infection. After treatment, the patient had no fever, no myalgia, no significant coughing, and a chest CT scan showed that pneumonia had been absorbed, inflammatory indexes were reduced, liver function improved, and he was discharged on August 2, 2022. According to the patient’s recollection, he had no history of contact with birds, other than a chicken he had killed in his hometown in Zhaoqing City, Guangdong Province, 1 week before the consultation. In particular, he had a smoking history of over 30 years, consuming 20 cigarettes per day, and had been consuming 2000–2500 mL of beer per occasion, 1–2 times per month, for many years.

### 2.2. Sample source and processing

Alveolar lavage fluid samples were collected from each of the patients in the cases described above (*n* = 3), and a total of 60 bird fecal samples were collected from four bird-selling stores in Shenzhen (Supplementary Material 3). Each of the 3 case patients came into contact with birds in different ways prior to becoming ill. For feasibility, and as common places of human-bird interaction, pet bird and poultry sales outlets were selected for sample collection in this study. In addition, to better illustrate the distribution pattern of *C. psittaci* and its hosts, we also screened the public database for representative sequences of different genotypes of *C. psittaci* and their hosts for comparative analysis (Supplementary Material 1). All samples were screened using a commercial kit (Aodong Inspection & Testing Technology Co., Ltd., Shenzhen, China) and subjected to nucleic acid extraction using DNeasy Blood & Tissues kit (Qiagen, Germany). The extracted nucleic acids from the patient’s alveolar lavage fluid were sent to Beijing Novozyme Biotech for sequencing.

### 2.3. Macrogenome data analysis

Sequencing data were filtered for possible human-derived reads using Bowtie2 software (version2.2.4).^1^ Sequence assembly was performed using MEGAHIT software (v1.0.4-beta). Scaftigs were assembled using MEGAHIT software (v1.0.4-beta) to obtain Clean Data. Scaftigs (≥500 bp) from each sample were predicted using MetaGeneMark (V2.10)^2^ and the predicted results were filtered out for lengths less than 100nt. For ORF prediction results, CD-HIT software (V4.5.8)^3^ was used for redundancy removal to obtain sequential nucleic acid sequences encoding genes. The Clean Data of each sample was compared to the initial gene catalog using Bowtie2, and the number of reads of the gene in each sample was calculated. From the reads and gene lengths, the abundance of each gene in each sample was calculated, and basic information, core-pan gene analysis, inter-sample correlation analysis, and Venn diagram analysis of gene numbers were performed. Species annotation was performed using Diamond software (V0.9.9.110),^4^ and Unigenes were aligned with bacterial (Bacteria), fungal (Fungi), archaeal (Archaea) and viral (Viruses) sequences extracted from NCBI’s NR database (Version 2018-01-02).^5^ Resistance Gene Analysis Unigenes were aligned to the CARD database^6^ using the Resistance Gene Identifier (RGI) software provided by the CARD database. The data from the macro-genome sequencing data were collated with the results of the CARD database comparison analysis and visualized using the circos online website^7^.

### 2.4. Genotyping

**FIGURE 1:**
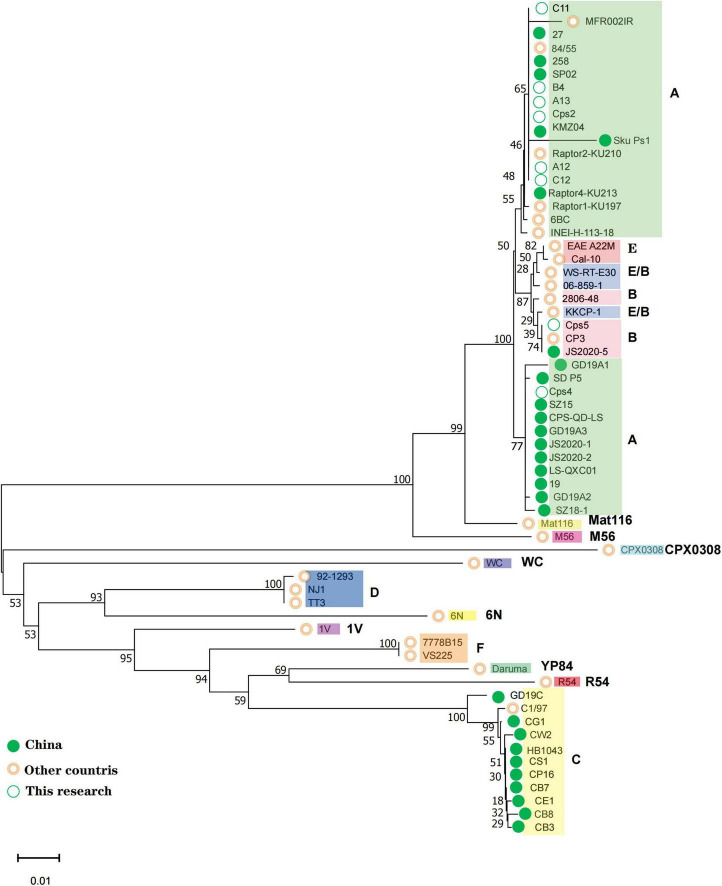
All positive samples of this study were compared with each genotype *ompA* gene sequence to the phylogenetic tree. The genotypes to which each sequence belongs are shown on the rightmost side, and the corresponding strains of each genotype are spaced by different color blocks. The green solid circle markers correspond to Chinese strains and the pink circles correspond to strains from other countries; the open green circles correspond to the three patient numbers and the five positive sample numbers from the bird-selling stores in this study.

Each sample nucleic acid was specifically amplified using 2X Pro Taq Master Mix (dye plus) kit (Acres Biotechnology Co., Ltd., Hunan, China) with primers using *ompA* specific primer sequences (Sachse et al., 2008). The amplification procedure was pre-denaturation 95°C for 2 min, denaturation 95°C for 30 s, annealing 55°C for 30 s, 35 cycles, extension 72°C for 1 min, and final extension 72°C for 2 min. PCR products were sent to Sangon Biotech (Shanghai, China) for two-way sequencing (Sanger). The sequences were spliced using SeqMan pro under DNASTAR Lasergene 11 software, compared with MegAlign. In this study, the amplified *ompA* sequences of *C. psittaci* were compared with the *ompA* core coding region sequences of known genotypes published by NCBI and representative *ompA* sequences in China and a phylogenetic tree was constructed (Figure 1 and Supplementary Material 1). The phylogenetic tree was constructed using Molecular Evolutionary Genetics Analysis software version MEGA X default parameters (Statistical Method: Neighbor-joining method; 1,000 bootstrap replicates; Substitutions p-distance model).

## 3. Results

### 3.1. Antibiotic use and treatment outcome comparison among the 3 patients

Patient in case Cps2 received 3 dosing regimens of sulphen (cephalosporin antibiotic), or meropenem (carbapenem antibiotic) combined with doxycycline (tetracycline antibiotic), or moxifloxacin (quinolone antibiotic) combined with terzicin (penicillin antibiotic) showed no significant improvement in symptoms. When *C. psittaci* infection was detected, the use of moxifloxacin in combination with doxycycline still did not show significant results. And then, treatment of with doxycycline with moxifloxacin and supplemental vancomycin combined with extracorporeal artificial membrane lung, resulted in symptomatic improvement. Cps4 was initially treated with cefoperazone sulbactam (cephalosporin antibiotic) and arbidol for pulmonary infection, which had no significant effect, and patients subsequently treated with doxycycline and azithromycin (macrolide antibiotic) showed significant improvement in symptoms. After no significant therapeutic effect with levofloxacin hydrochloride (quinolone antibiotic), Cps5 was switched to cefprozil granules (cephalosporin antibiotic) and doxycycline tablets, and the patient’s symptoms were significantly improved.

### 3.2. Macrogenomic analysis of patient samples

**FIGURE 2:**
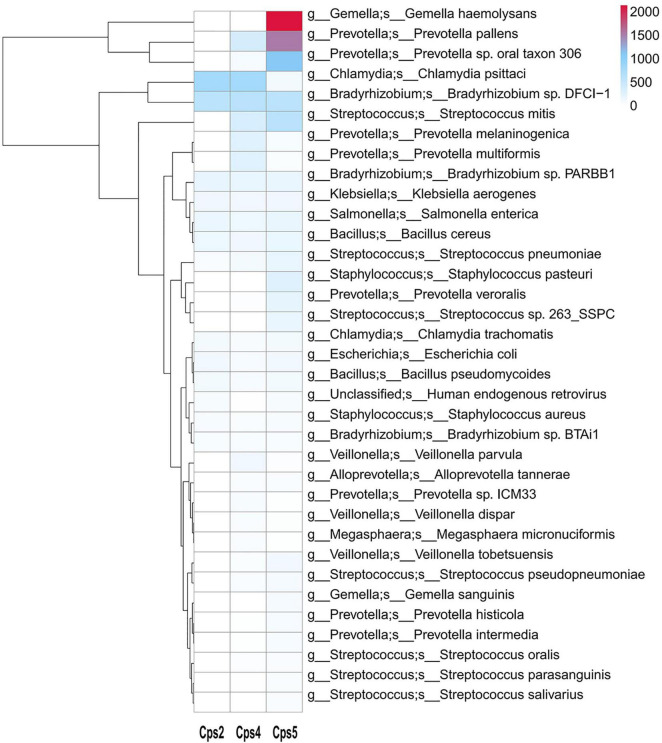
Heatmap of microbial species levels based on gene numbers in samples from three patients. This species annotation heatmap from the macrogenome sequencing data. The *x* axis shows the sample number, and the secondary *y* axis shows the abundance of microorganisms in each sample, as indicated by the color key. Cluster analysis clusters species according to their abundance distribution in the sample, and species clustered together have a similar “abundance” distribution in the sample.

**FIGURE 3:**
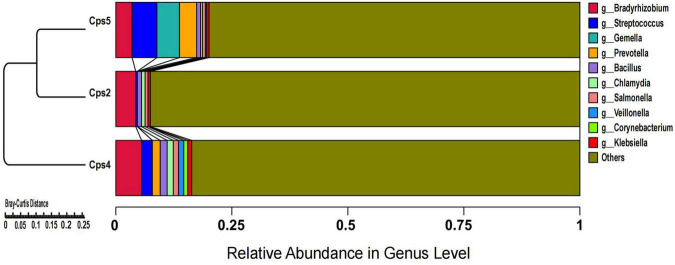
Cluster analysis of microbial genes at the genus level after de-hosting sequences of samples from three patients.

**FIGURE 4:**
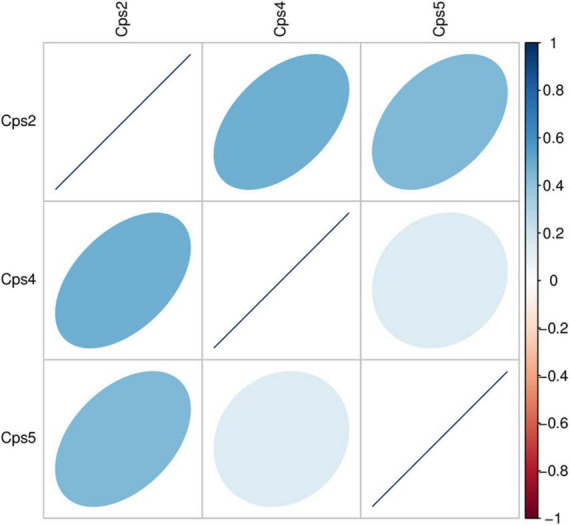
Heatmap of correlation coefficient of gene abundance between samples. The heatmap displays the level of Spearman correlation coefficients between different samples, with each color representing a different level of correlation. The legend on the right explains the relationship between the colors and correlation coefficients. A darker color indicates a larger value of absolute correlation coefficient. The orientation of the ellipse indicates whether the correlation coefficient is positive (ellipsoid tilts toward the right) or negative (ellipsoid tilts toward the left). A flatter ellipse indicates a larger absolute value of correlation coefficient.

**FIGURE 5:**
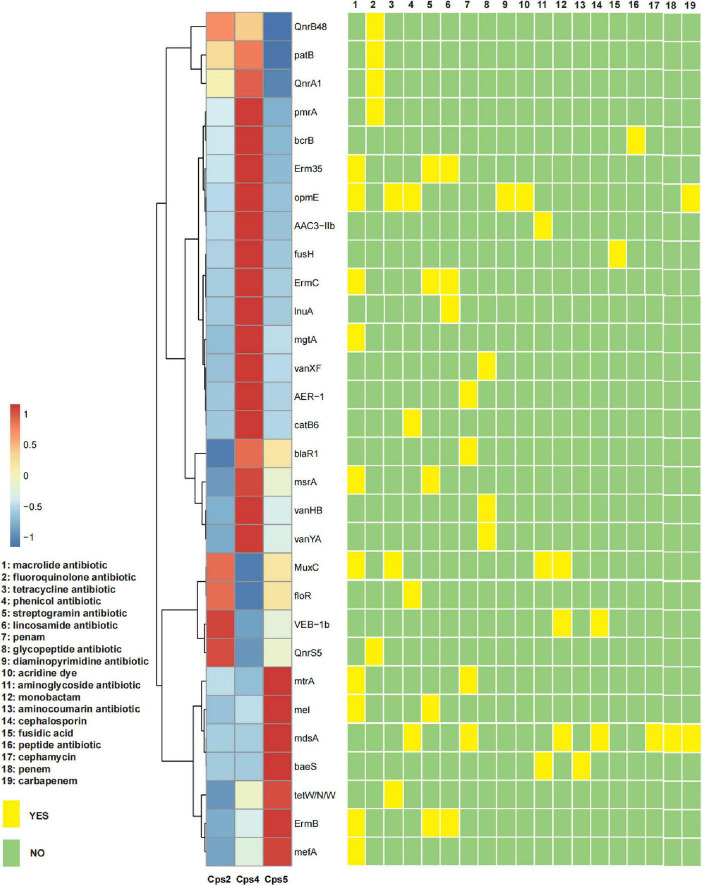
Heatmap of the abundance of the top 30 antibiotic resistance genes for each sample and their corresponding targeted drug classes. The *x* axis of the heatmap is the sample number, and the *y* axis is the name of the antibiotic resistance gene; a redder color block indicates a higher abundance of the antibiotic resistance gene, and a bluer color block indicates a lower abundance of the antibiotic resistance gene, as shown in the top left legend (values were obtained by normalized conversion). The yellow and green squares on the right indicate whether the corresponding antibiotic resistance gene is targeted to a certain class of antibiotics, and the antibiotic class is detailed in the legend on the left.

**FIGURE 6:**
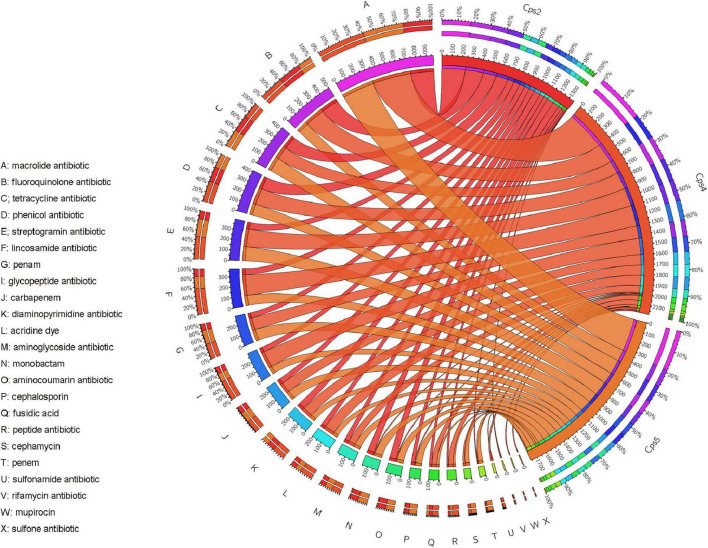
Circos plot of different resistance gene abundance for each sample with corresponding resistance gene phenotypes. The data from the macro-genome sequencing data were collated with the results of the CARD database comparison analysis and visualized using the circos online website (http://mkweb.bcgsc.ca/tableviewer/). The letters on the left side of the circos plot represent each type of antibiotic, corresponding to the annotated information on the left; the sample numbers of the three patients are shown on the right side of the plot. The module corresponding to the sample number is the relative percentage of antibiotic resistance genes contained in that sample targeted against each drug class, distinguished by different colors in the outer circle. The module corresponding to the letter indicates the relative percentage of antibiotic resistance gene abundance targeting that class of antibiotics in the three samples. The inner circle is the abundance or number of antibiotic resistance genes.

**FIGURE 7:**
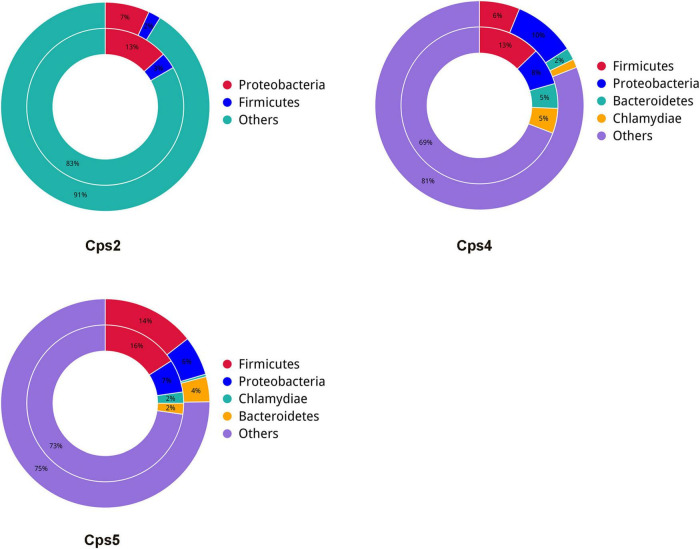
Bicircle plot of antibiotic resistance genes in relation to species affiliation at the phylum level for each patient sample. Based on the gene abundance in each sample, the inner circle indicates the distribution of species to which the resistance gene belongs in that sample, and the outer circle shows the distribution of species to which all genes in that sample belong.

Macrogenomic analysis data of all three patients showed *C. psittaci* infection. Comparative analysis of the number of *C. psittaci* annotated genes presented showed Cps2 and Cps4 with similar proportions at 2.38% (805/33787) and 2.23% (805/36165), respectively, and Cps5 with a lower proportion at 0.22% (109/49011) (Figure 2). Some genes also matched to *C. trachomatis* (102, 70, 108) or *C. pneumoniae* (1, 1, 0) (Figures 2, 3, fewer sequences of *C. pneumoniae* are not shown in the figure). Among the total genes in each sample, the similarity between the genes contained in Cps2 and Cps4 or Cps5 was higher, and the similarity between the genes contained in Cps4 and Cps5 was lower (Figure 4). However, among the annotatable species compositions, Cps2 and Cps5 were more similar at the genus level (Figure 3). Different proportions of antibiotic resistance genes were present in each sample, and mainly targeted macrolide antibiotics, fluoroquinolone antibiotics, tetracycline antibiotics, chloramphenicol antibiotics, and streptococcal antibiotics (Figures 5, 6). Antibiotic resistance genes were present in *Chlamydomonas* in both Cps4 and Cps5, accounting for 5 and 2% of the antibiotic resistance genes in their respective samples (Figure 7). A resistance gene, *mupB*, that exactly matched the sequence of *C. psittaci* (1. GenBank: EPJ31254.1. strain: 06-1683; host: *Homo sapien*; country: France, 2. GenBank: WP_014944502.1, 3. GenBank: EPP32097.1; strain: C1/97; host: *Ovis aries*; country: Germany), was present in Cps4, and the drug resistance gene *OprM*, which exactly matched the sequence of *C. psittaci* (GenBank: EPJ27639.1; strain: 99DC5; host: *Equus caballus*; country: Germany), was present in both Cps4 and Cps5. According to the data in the CARD database the main antibiotic targeted by the *mupB* gene is mupirocin, while the *OprM* gene targets multiple antibiotics.

### 3.3. Genotype analysis

*C. psittaci* was detected in 5 (A12, A13, B4, C11, C12) out of 60 bird fecal samples collected from bird-selling stores. The near-complete *ompA* gene was successfully amplified with sequence lengths of Cps2-1120 bp, Cps4-1142 bp, Cps5-1128 bp, A12-1125 bp, A13-1129 bp, B4-1121 bp, C11-1049 bp, C12-1129 bp from the 3 patient samples (Cps2, Cps4, Cps5) and 4 bird fecal samples. In the constructed phylogenetic tree, using known sequences of 15 genotypes (A, B, C, D, E, F, E/B, M56, WC, Mat116, CPX308, 6N, 1V, R54, YP84), it was found that the A, B, E, and E/B types were more similar and therefore more difficult to distinguish (Figure 1). Our samples fell in the middle of these 4 genotypes and so the amino acid sequences of each genotype were compared and analyzed. It was found that the genotype A differed from the other genotypes mainly by the amino acid isoleucine (I) at position 170, while the B, E and E/B genotypes differed from the other genotypes mainly by the amino acid serine (S) at positions 151 and 152. The E genotype differed from other genotypes in amino acids 336 and 337 for threonine (T) and leucine (L), respectively. The B and E/B genotypes differed in amino acid 229 and 341. While the amino acid at position 229 of the genotype E/B is valine (V) or isoleucine (I) and position 341 is alanine (A) or serine (S), the amino acids at the two loci did not both correspond to the equivalent amino acids of genotype B (Supplementary Material 2). Our samples were consistent with the specific sites of the amino acid sequence of genotype A, except for Cps5, which was consistent with the specific sites of the amino acid sequence of genotype B. Interestingly, we found that in a clustering analysis based on different genotypes *ompA* gene sequences, *C. psittaci* of genotype A was divided into two clusters, one with a host source of mainly birds and the other with a host of mainly poultry such as ducks and geese.

## 4. Discussion

In our study, all three patients presented with clinical symptoms previously reported for psittacosis, such as high fever, malaise, and lesions in the lungs (Zhang et al., 2021). According to the analysis of resistance gene abundance and corresponding resistance drugs, it was found that the relative proportion of resistance drugs corresponding to the resistance genes of microorganisms in the alveolar lavage fluid of the three patients was similar, and the drugs mainly targeted were macrolide antibiotics, fluoroquinolone antibiotics, tetracycline antibiotics, chloramphenicol antibiotics, and streptococcal antibiotics in that order (Figure 6). We also noted a high abundance of respiratory or gastrointestinal opportunistic pathogens, such as *Gemella haemolysans*, *Prevotella pallens*, and *Prevotella* sp. oral taxon 306, in the alveolar lavage fluid sample from patient Cps5 (Figure 2). These bacteria are usually present in the upper respiratory tract of humans. Among them, *Gemella haemolysans* is one of the clinically significant opportunistic pathogens that can cause inflammatory lesions in soft tissues of immunocompromised patients, such as endocarditis, pneumonia, and meningitis (García-Lechuz et al., 2002; Ernesto and Martín-Galiano, 2020; Eslinger and Ahmed, 2022). According to clinical reports, Gemella is usually susceptible to penicillins, ceftriaxone, and vancomycin (Chesdachai et al., 2022). Combined with the patient’s Cps5 medication history, taking cephalosporin antibiotics may have been a factor in the improvement of his clinical symptoms. Based on the clinical history of the three patients, we found that patient Cps5 had a history of smoking and alcohol consumption for many years, which is different from the other two patients. Prolonged smoking may lead to the displacement of the upper respiratory tract microbiota and make a person more susceptible to opportunistic pathogens, resulting in the development of the disease (Hilty et al., 2020; Jia et al., 2021; Fleischmann et al., 2023).

Patients Cps4 and Cps5 showed symptomatic improvement with doxycycline treatment, however, patient Cps2, though treated with doxycycline or moxifloxacin, did not have significant symptomatic improvement. Cps2’s subsequent treatment of with doxycycline with moxifloxacin and supplemental vancomycin combined with extracorporeal artificial membrane lung, resulted in symptomatic improvement. In other studies, clinical symptoms of the patients given moxifloxacin or cephalosporin antimicrobial therapy in early treatment also failed to improve (Zhang et al., 2020, 2021). Similarly, doctors administered tetracycline, macrolides, and fluoroquinolones simultaneously to treat a patient with severe parrot fever before sufficient improvement for discharge after 28 days (Shi et al., 2021). The conventional dose of medication for the clinical treatment of some patients with severe parrot fever may not be sufficient to achieve the therapeutic purpose, and it therefore should be adjusted according to the patient’s condition (Wang et al., 2022). *In vitro* studies have shown that 50 U/ml penicillin has no significant inhibitory effect on the activity and infectivity of *C. psittaci*, but 100 U/ml penicillin can maintain it in a viable but non-infected state (Hu et al., 2015). The above study suggests the presence of antibiotic resistance in *C. psittaci*. Microbial antibiotic resistance genes have a non-negligible share in the data from our patient’s macrogenome sequencing (mNGS), and among them genes *mupB* and *OprM* match *C. psittaci*, and may be resistant to mupirocin and multiple antibiotics, respectively. Previous studies suggest that *C. psittaci* may be resistant to aminoglycosides through mutations in the *16S rRNA* and *rpoB* and to macrolides through mutations in the *23S rRNA* (Benamri et al., 2021). *Chlamydia* spp. can also acquire drug resistance genes from external sources (Marti et al., 2022), as demonstrated by Dugan et al. (2004), with *C. porcine* able to acquire tetracycline-resistant genes through horizontal gene transfer. It has been suggested that prophylactic use of tetracycline may induce *Chlamydia*-acquired tetracycline resistance (Vanrompay et al., 2007; Bommana and Polkinghorne, 2019; Zhang et al., 2021). Thus, *Chlamydia* spp. can acquire antibiotic resistance through its own genetic mutations, acquisition of resistance genes, and environmental induction. As the causative strains of patients’ psittacosis were not obtained, the antibiotic resistance mechanism of *C. psittaci* in this study could not be validated.

The use of mNGS in the three patient cases enabled rapid screening for pathogenic organisms, leading to targeted and effective treatment, and offering significant advantages over the common methods currently used for pathogen detection (Zhang et al., 2021). For example, the common methods of analysis of body fluid smears or cultures using enzyme-linked immunosorbent assay (ELISA) or polymerase chain reaction (PCR) are influenced by multiple factors, are sensitive to contamination, often provide false-positive results and make it difficult to detect multiple pathogens simultaneously (Duan et al., 2022). Similarly, the use of isolated cultures to detect pathogens such as *Chlamydia*, *Mycobacterium tuberculosis*, *Mycoplasma*, *Legionella*, *Cryptococcus*, *fungi*, *viruses*, and *Pneumocystis* not only takes a long time, but also has a low positivity rate and is therefore not conducive to rapid clinical diagnoses. In contrast, the use of mNGS testing in the identification of pathogens in patients with severe community-acquired pneumonia (SCAP) offers significant advantages (Qu et al., 2022), such as that it is not affected by non-pathogenic bacteria causing disease and early antibiotic use. The high sensitivity and detection rates of mNG also present significant advantages in the diagnosis of unknown, specific, and drug-resistant pathogens, providing results within 48–72 h (Lavezzo et al., 2016; Deurenberg et al., 2017). However, mNGS also has limitations in that it is more difficult to analyze the complete whole genome sequence of a single pathogenic bacterium in samples with a high percentage of host sequences, and when only partial or small sequences of the bacterium can be obtained. Furthermore, huge storage capacity and specialized bioinformatics analysts are required to manage and analyze the data. In this study, although the sequence of *C. psittaci* was dominant in the genus *Chlamydia*, *C. trachomatis* and a small number of *C. pneumoniae* genes were also found, potentially due to differential gene similarity within the genus *Chlamydia* (Voigt et al., 2012; Radomski et al., 2016).

Companion birds and poultry are the most important sources of *C. psittaci* infection in patients, but some patients, including patient Cps4, do not have a history of direct contact with live birds or poultry (Balsamo et al., 2017; Hogerwerf et al., 2020; Duan et al., 2022; Liu and Gao, 2022). In northwest China, the prevalence of *C. psittaci* in marketed poultry was 13% in chickens, 39% in ducks, and 31% in pigeons (Cong et al., 2013). Our samples of C12, B4, A13, A12, and Cps2 that belonged to genotype A, were all associated with parrots and clustered together with the *ompA* sequence of the earliest genotype A strain 6BC. Patient sample Cps4, with unknown patient exposure history, clustered differently, and with sequences previously identified as genotype A and derived from ducks or geese in Shandong, Guangdong, Gansu, Jiangsu, and Beijing, China. Combined with the patient’s self-reported exposure history, it is assumed that her most likely route of infection was contact with poultry at the time of the food market purchase. Patient sample Cps5, belonged to genotype B, clustered in another cluster with the *ompA* sequence of strain JS2020-5 from Nanjing, Jiangsu, China.

Zhang Z. et al. (2022) analyzed the transmission chain of *C. psittaci* and found the causative strain to be genotype A (SD_P5, MZ345290.1), with most patients having close associations with a duck processing plant and experienced human-to-human transmission. Similarly, human-to-human transmission of *C. psittaci* genotype A associated with parrots was reported in Sweden (Wallensten et al., 2014; Zhang Z. et al., 2022). Genotype A strain (SZ15, MK630234.1) has also been isolated from ducks, in contrast to previous reports of *C. psittaci* genotype C and genotype E/B being isolated from ducks (Lin et al., 2019). *C. psittaci* genotype C has also been previously detected in pigs, cattle, sheep, wild birds, and ducks, differing from the accepted avian origin (Song et al., 2009). In our study, the exposure history of patient Cps5 suggests that the source of the infection may have been chickens, which is also different from previous reports that genotype B originated from pigeons (Geens et al., 2005). Consistently, sequence comparison among genotypes showed that the same genotype sequence is relatively conserved but can still differ and has a certain evolutionary pattern (Supplementary Material 2). This suggests that pathogenic genotypes may be transmitted by multiple hosts, and that genotypes and hosts do not correspond uniquely, but the correspondence between genotypes and hosts is relatively stable in a certain period or region. This genotypic diversity and host variation reflects the adaptability of *C. psittaci* to the host or environment (Bush and Everett, 2001).

## 5. Conclusion

Macrogenomic sequencing (mNGS) is an effective and rapid detection tool for difficult-to-identify pathogens in clinical cases, and macrogenomic data can provide insight into the sequence data of pathogenic bacteria. Although we could not obtain the complete genomic data of *C. psittaci*, we found antibiotic resistance genes belonging to *C. psittaci* in patients Cps4 and Cps5. Meanwhile, the medication of three patients and other clinical cases indicate that *C. psittaci* is currently resistant to some antibiotics and its resistance genes may have been altered. Furthermore, the genotype and host of *C. psittaci* are related, with coding sequences of genotypes in the same region relatively conserved and with relatively stable hosts, which may be used to facilitate the traceability and prevention and control of *C. psittaci* in the future.

## Data availability statement

The data presented in the study are deposited in the NCBI repository under BioProjects PRJNA953446, PRJNA956132, and PRJNA954677.

## Ethics statement

The studies involving human participants were reviewed and approved by the Ethics Committee of the Shenzhen Center for Disease Control and Prevention (QS2023040054). Written informed consent from the participants’ legal guardians/next of kin was not required to participate in this study, in accordance with the national legislation and the institutional requirements.

## Author contributions

HL and XS conceived and designed the study. JY, ZW, and LW collected the case-related data. LJ, SQ, and SX collected the samples of bird droppings from pet stores. SW, QC, ZZ, ZC, XZ, QH, TF, JL, MJ, YL, BP, QB, RC, and YG carried out the experiments and data analysis. HL drafted the manuscript. XS revised the manuscript. HL, JY, ZW, and LW have the same contribution to this work. All authors have read and agreed to the published version of the manuscript.

## Footnotes

1
http://bowtie-bio.sourceforge.net/bowtie2/index.shtml

2
http://exon.gatech.edu/GeneMark/

3
http://www.bioinformatics.org/cd-hit/

4
https://github.com/bbuchfink/diamond/

5
https://www.ncbi.nlm.nih.gov/

6
https://card.mcmaster.ca/

7
http://mkweb.bcgsc.ca/tableviewer/
