# Supplementary material for: Distribution of drug-resistant genes in alveolar lavage fluid from patients with psittacosis and traceability analysis of causative organisms
Source: Front Microbiol. 2023 Jun 22;14:1182604. doi: 10.3389/fmicb.2023.1182604 (PMC10327639; doi:10.3389/fmicb.2023.1182604)
Supplement: Supplementary file 1 [file Data_Sheet_1.zip › Supplementary Material 2.pdf]

+ Majority

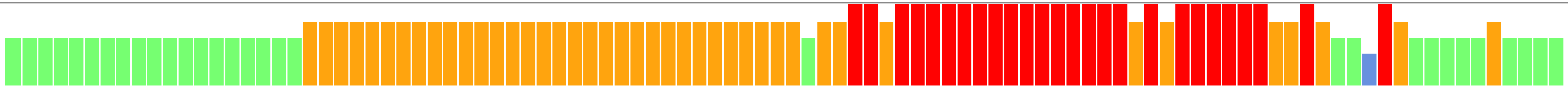

```
Majority          MKKLLKSALLFAATGSALSLQALPVGNPAEPSLLIDGTMWEGASGDPCDPCATWCDAISIRAGYYGDYVFDRVLKVDVNKTFSGMAATPTQATGNASNTN
                           10        20        30        40        50        60        70        80        90       100
C11               XXXXXXXXXXXXXXXXXXXXXXXXXXXXXXXXXXXXXX..............................................................  62
MFR002IR          ---------------------------------------.............................................................  61
27                XXXXXXXXXXXXXXXXXXXXXXXXXXXXXXX.....................................................................  69
84-55             ....................................................................................................  100
258               XXXXXXXXXXXXXXXXXXXXXXXXXXX.........................................................................  73
SP02              ....................................................................................................  100
B4                ----------------....................................................................................  84
A13               XXXXXXXXXXXXXX......................................................................................  86
Cps2              XXXXXXXXXXXXXXXXX...................................................................................  83
KMZ04             ....................................................................................................  100
Sku_Ps1           ....................................................................................................  100
Raptor2-KU210     ----------------------..............................................................................  78
A12               XXXXXXXXXXXXXXX.....................................................................................  85
C12               XXXXXXXXXXXXXX......................................................................................  86
Raptor4-KU213     ----------------------..............................................................................  78
Raptor1-KU197     ----------------------..............................................................................  78
6BC               ....................................................................................................  100
INEI-H-113-18     --------------......................................................................................  86
EAE_A22M          ....................................................................................................  100
Cal-10            ....................................................................................................  100
WS-RT-E30         ....................................................................................................  100
06-859-1          XXXX................................................................................................  96
2806-48           .......................................................................................A............  100
KKCP-1            ............V.......................................................................................  100
Cps5              ----------------....................................................................................  84
CP3               ....................................................................................................  100
JS2020-5          XXXXXXXXXXXXXXXXXXXXXXXXXXXX........................................................................  72
GD19A1            .......................................................................................A............  100
SD_P5             .......................................................................................A............  100
Cps4              XXXXXXXXXXXXX..........................................................................A............  87
SZ15              .......................................................................................A............  100
CPS-QD-LS         .......................................................................................A............  100
GD19A3            .......................................................................................A............  100
JS2020-1          XXXXXXXXXXXXXXXXXXXXXXXXXXX............................................................A............  73
JS2020-2          XXXXXXXXXXXXXXXXXXXXXXXXX..............................................................A............  75
LS-QXC01          .......................................................................................A............  100
19                .......................................................................................A............  100
```

GD19A2	....A............	100
SZ18-1	....A.S..........	100
Mat116	....S....................I.........T.......EP.......G	100
M56	....S..............................T.......E.S...T..G	100
CPX0308	....S.............................V.....A..G.S.T..G.X	100
WC	....I..ESS.TV.SAX	100
92-1293	....V...KS..E...T..A.T	100
NJ1	XX..KS..E...T..A.T	98
TT3	....KS..E...T..A.T	100
6N	XXXXXXXXXXXXXXXXXXX................................S....................I.........T.........S.T....X	81
1V	XXXXXXXXXXXXXXXXXXXXXXXXX..........................S.................................GKA..GNXXT.EDFX	73
7778B15	....I...G.A..GSXXA.ADYX	98
VS225	....I...G.A..GSXXA.ADYX	98
Daruma	....S..............................T..G.V..GNXXS.ADFX	98
R54	XXXXXXXXXXXXXXXXXXXXXXXXXXXXXXXXXXXXXXXXXXXXXXXXXXXXXX...........................I...GKA..GSXXA.QDYX	44
GD19C	....S................................IGKK..GSXXSPNDFX	98
C1-97	....S................................IGKK..GSXXSPNDFX	98
CG1	....S................................IGKK..GSXXSPNDFX	98
CW2	.............................G.....................S................................IGKK..GSXXSPNDFX	98
HB1043	....S................................IGKK..GSXXSPNDFX	98
CS1	....S................................IGKK..GSXXSPNDFX	98
CP16	....S................................IGKK..GSXXSPNDFX	98
CB7	....S................................IGKK..GSXXSPNDFX	98
CE1	....S................................IGKK..GSXXSPNDFX	98
CB8	....S......................E.........IGKK..GSXXSPNDFX	98
CB3	....S....T...........................IGKK..GSXXSPNDFX	98

+ Majority

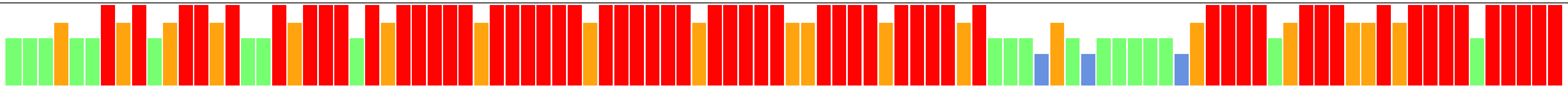

```
Majority        QPEANGRPNI AYGRHMQDAE WFSNAAFLAL NIWDRFDIFC TLGASNGYFK ASSAAFNLVG LIGFSATSSI STDLPTQLPN VGITQGVVEF YTDTSFSWSV
                       110        120        130        140        150        160        170        180        190        200

C11             .......... ......E... .......... .......... .......... .......... ......A... .......... .......... ..........  162
MFR002IR        .......... ......E... .......... .......... .......... .......... ......A... .......... .......... ..........  161
27              .......... ......E... .......... .......... .......... .......... ......A... .......... .......... ..........  169
84-55           .......... ......E... .......... .......... .......... .......... ......A... .......... .......... ..........  200
258             .......... ......E... .......... .......... .......... .......... ......A... .......... .......... ..........  173
SP02            .......... ......E... .......... .......... .......... .......... ......A... .......... .......... ..........  200
B4              .......... ......E... .......... .......... .......... .......... ......A... .......... .......... ..........  184
A13             .......... ......E... .......... .......... .......... .......... ......A... .......... .......... ..........  186
Cps2            .......... ......E... .......... .......... .......... .......... ......A... .......... .......... ..........  183
KMZ04           .......... ......E... .......... .......... .......... .......... ......A... .......... .......... ..........  200
Sku_Ps1         .......... ......E... .......... .......... .......... .......... ......A... .......... .......... ..........  200
Raptor2-KU210   .......... ......E... .......... .......... .......... .......... ......A... .......... .......... ..........  178
A12             .......... ......E... .......... .......... .......... .......... ......A... .......... .......... ..........  185
C12             .......... ......E... .......... .......... .......... .......... ......A... .......... .......... ..........  186
Raptor4-KU213   .......... .......... .......... .......... .......... .......... ......A... .......... .......... ..........  178
Raptor1-KU197   .......... .......... .......... .......... .......... .......... ......A... .......... .......... ..........  178
6BC             .......... .......... .......... .......... .......... .......... ......A... .....M.... .......... ..........  200
INEI-H-113-18   .......... .......... .......... .......... .......... .......... ......A... .......... .D........ ..........  186
EAE_A22M        .......... .......... .......... .......... .......... S......... .........T ..E..M.... .......... ..........  200
Cal-10          .......... .......... .......... .......... .......... S......... .........T ..E..M.... .......G.. ..........  200
WS-RT-E30       .......... .......... .......... .......... .......... S......... .........T ..E..M.... .......... ..........  200
06-859-1        .......... .......... .......... .......... .......... S......... .........T .....M.... .......... ..........  196
2806-48         .......... .......... .......... .......... .......... S......... .........T .....M.... .......... ..........  200
KKCP-1          .......... .......... .......... .......... .......... S......... .......N.T .A...M.... .......... ..........  200
Cps5            .......... .......... .......... .......... .......... S......... .......N.T .....M.... .......... ..........  184
CP3             .......... .......... .......... .......... .......... S......... .......N.T .....M.... .......... ..........  200
JS2020-5        .......... .......... .......... .......... .......... S......... .......N.T .....M.... .......... ..........  172
GD19A1          .......... .......... .......... .......... .......... .......... .......... .......... .......... ..........  200
SD_P5           .......... .......... .......... .......... .......... .......... .......... .......... .......... ..........  200
Cps4            .......... .......... .......... .......... .......... .......... .......... .......... .......... ..........  187
SZ15            .......... .......... .......... .......... .......... .......... .......... .......... .......... ..........  200
CPS-QD-LS       .......... .......... .......... .......... .......... .......... .......... .......... .......... ..........  200
GD19A3          .......... .......... .......... .......... .......... .......... .......... .......... .......... ..........  200
JS2020-1        .......... .......... .......... .......... .......... .......... .......... .......... .......... ..........  173
JS2020-2        .......... .......... .......... .......... .......... .......... .......... .......... .......... ..........  175
LS-QXC01        .......... .......... .......... .......... .......... .......... .......... .......... .......... ..........  200
19              .......... .......... .......... .......... .......... .......... .......... .......... .......... ..........  200
```

```
GD19A2     ..............................S.....................................................................  200
SZ18-1     ..........V.........................................................................................  200
Mat116     T..................................................................T.T..............................  200
M56        T......A..........................................................S.AV.....K.....A..................  200
CPX0308    XXTPVE...L...K........T..............V......T.....SN....S......VAGG.LNXXXXXNE....FM...I..L....T.....  194
WC         XXQ.VD.V.L...K.L......T.S.........................G..........F.IAGN.ESNAX.ND.....A....I.......T.....  197
92-1293    XXT.VD.T.L...K.L......T........................................LKG.DFNXXXXXN.....A............T.....  193
NJ1        XXT.VD.T.L...K.L......T........................................LKG.DFNXXXXXN.....A............T.....  191
TT3        XXT.VD.T.L...K.L......T........................................LKG.DFNXXXXXN.....A............T.....  193
6N         XXT.AD.T.L...K.L......T.....................A......G...........VAGSDLNXXXXXN.....A............T.....  174
1V         XXI.ED.........L......T........................................VKG..VDAAX.N...................T.....  170
7778B15    XXTPTD.......K.L......T........................................VKG..VAXXXXXD..........I.......T.....  192
VS225      XXTPTD.......K.L......T........................................VKG..VAXXXXXD..........I.......T.....  192
Daruma     XXTPTD.A.....K.L......T........................................VKGNTLTXXXXXD..................T.....  192
R54        XXA.ED.A.....K.L......T........................................VKGA.LSXXXXXE..................T.....  137
GD19C      XXN.ED...V.....L..S...T........................................VKGS.LTXXXXXD.....A............ .....  192
C1-97      XXN.ED...V.....L..S...T........................................VKGS.LTXXXXXD.....A............T.....  192
CG1        XXK.ED...V.....L..S...T........................................VKGN.LTXXXXXD.....A............T.....  192
CW2        XXN.ED...V.....L..S...T........................................VKGN.LTXXXXXD.....A............T.....  192
HB1043     XXN.ED...V.....L..S...T........................................VKGN.LTXXXXXD.....A............T.....  192
CS1        XXN.ED...V.....L..S...T........................................VKGN.LTXXXXXD.....A............T.....  192
CP16       XXN.ED...V.....L..S...T........................................VKGN.LTXXXXXD.....A............T.....  192
CB7        XXN.ED...V.....L..S...T........................................VKGN.LTXXXXXD.....A............T.....  192
CE1        XXN.ED...V.....L..S...T........................................VKGN.LTXXXXXD.....A............T.....  192
CB8        XXN.ED...V.....L..S...T........................................VKGN.LTXXXXXD.....A............T.....  192
CB3        XXN.ED...V.....L..S...T........................................VKGN.LTXXXXXD.....A............T.....  192
```

+ Majority

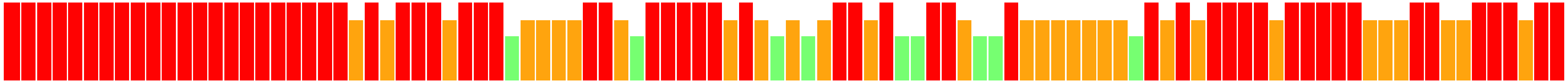

Majority	GARGALWECGCATLGAEFQYAQSNPKIEMLNVTSSPAQFVIHKPRGYKGASSNFPLPITAGTTEATDTKSATIKYHEWQVGLALSYRLNMLVPYIGVNWS	
	210 220 230 240 250 260 270 280 290 300	
C11	....	262
MFR002IR	....HN......	261
27	....	269
84-55	....	300
258	....	273
SP02	....	300
B4	....	284
A13	....	286
Cps2	....	283
KMZ04	....	300
Sku_Ps1	....L...I.........SG.N.S.....D..........SIE..........	300
Raptor2-KU210	....	278
A12	....	285
C12	....	286
Raptor4-KU213	....	278
Raptor1-KU197	....	278
6BC	....	300
INEI-H-113-18	....	286
EAE_A22M	............................V...	300
Cal-10	............................V...	300
WS-RT-E30	............................V...	300
06-859-1	............................I...	296
2806-48	............................I...	300
KKCP-1	............................V...	300
Cps5	............................I...	284
CP3	............................I...	300
JS2020-5	............................I...	272
GD19A1	....	300
SD_P5	....	300
Cps4	....	287
SZ15	....	300
CPS-QD-LS	....	300
GD19A3	....	300
JS2020-1	....	273
JS2020-2	....	275
LS-QXC01	....	300
19	....	300

GD19A2	....	300
SZ18-1	....	300
Mat116	................................I.............................A..I..................................	300
M56	........................R........................T............DD....................................	300
CPX0308	............................T...ICNT...A........XIKXX....LN...V........I.........M...............K..	292
WC	....TT......L....DT....................................	297
92-1293	....TG.......D...EA........L...........................	293
NJ1	....TG.......D...EA........L...........................	291
TT3	....TG.......D...EA........L...........................	293
6N	......................F.......................S..T.W.....L....DT.....F..L.F......Q..................	274
1V	................................X.......V........T.......L....DQ...I...I.................R..........	269
7778B15	................................I...T...V........TG......L....DG........L...........................	292
VS225	................................I...T...V........TG......L....DG........L...........................	292
Daruma	....V.........AA.....E...SDA........L...........................	292
R54	................................V.......V........TTA.....L....DT........L...........................	237
GD19C	................................I.......V........T.A.....AN...EA........L...........................	292
C1-97	................................I.......V........T.A.....AN...EA........L...........................	292
CG1	................................I.......V........T.A.....AN...EA........L...........................	292
CW2	................................I.......V........T.A.....AN...EA........L...........................	292
HB1043	................................I.......V........T.A.....AN...EA........L...........................	292
CS1	................................I.......V........T.A.....AN...EA........L...........................	292
CP16	................................I.......V........T.A.....AN...EA........L...........................	292
CB7	................................I.......V........T.A.....AN...EA........L...........................	292
CE1	................................I.......V........T.A.....AN...EA........L........................S..	292
CB8	................................I.......V........T.A.....AN...EA........L...........................	292
CB3	................................I.......V........T.A.....AN...EA........L...........................	292

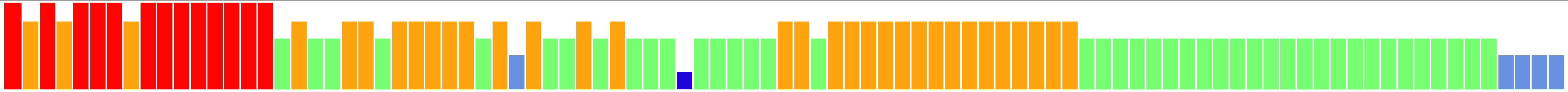

```
+ Majority
Majority        RATFDADTIRIAQPKLKSEILNITTWNPSLIGSTTALPNNSGKDVLSDVLQIASIQINKMKSRKACGVAVGATLIDADKWSITGEARLINERA-------
                         310       320       330       340       350       360       370       380       390       400
C11             .......................................................................................              350
MFR002IR        ...........................................G..M                                                   308
27              ................................................................X                                    334
84-55           .............................................................................................AHMNAQF 400
258             ..................................................................                                   340
SP02            .............................................................................................AHMNAQF 400
B4              .........................................................................................K           374
A13             .........................................................................................X           377
Cps2            .........................................................................................X           374
KMZ04           .............................................................................................AHMNAQF 400
Sku_Ps1         .............................................................................................AHMNAQF 400
Raptor2-KU210   ..................................................                                                   328
A12             ........................................................................................SX           375
C12             .........................................................................................X           376
Raptor4-KU213   ..................................................                                                   328
Raptor1-KU197   ..................................................                                                   328
6BC             .............................................................................................AHMNAQF 400
INEI-H-113-18   ........................................G..........M                                               338
EAE_A22M        ..............................L....T....G....................................................AHMNAQF 400
Cal-10          ..............................L....T....G....................................................AHMNAQF 400
WS-RT-E30       ..............................L.........A....................................................AHMNAQF 400
06-859-1        ..............................L.........A........X                                                   346
2806-48         ..............................L..............................................................AHMNAQF 400
KKCP-1          ..............................L..............................................................AHMNAQF 400
Cps5            ..............................L..........................................................KK.         376
CP3             ..............................L.................................S............................AHMNAQF 400
JS2020-5        ..............................L.......................................                               342
GD19A1          ........................................A....................................................AHMNAQF 400
SD_P5           ........................................A....................................................AYMNAQF 400
Cps4            ........................................A....................................................        381
SZ15            ........................................A....................................................AHMNAQF 400
CPS-QD-LS       ........................................A....................................................AHMNAQF 400
GD19A3          ........................................A....................................................AQMKAQF 400
JS2020-1        ........................................A................................X                           347
JS2020-2        ........................................A..........................                          343
LS-QXC01        ........................................A....................................................AHMNAQF 400
19              ........................................A....................................................AQMKAQF 400
```

```
GD19A2    ........................................A....................................................AHMNAQF  400
SZ18-1    ........................................A....................................................AHMNAQF  400
Mat116    ........................................G....................................................AHMNAQF  400
M56       ................................................................S............................AHMNAQF  400
CPX0308   .......S........ATA...L.A...T.L..A.SVXXXD.TNKF..FM..V.M.VD...........I...................D...AHLNAQF  389
WC        ................ATAV.DAK....TIT.ASGSVXXXDNTNKW..N............................................AHMNAQF  394
92-1293   .T..............ATAV.DL.....T.L.KA.TVXXXD.TNTY..F..L..............                                  356
NJ1       ................ATAV.DL.....T.L.KA.TVXXXD.TNTY..F..L................................D....H...AHMNAQF  388
TT3       ................ATAV.DL.....T.L.KA.TVXXXD.TNTY..F..L.........................................AHMNAQF  390
6N        ...Y............ATAV.DL.....T.L.KA.SVXXXG.TNIYX                                                     318
1V        ................ATAVX                                                                              290
7778B15   .......S........AAAV..L.....T.L.EA..XXXXDASNKFC.F..............                                    355
VS225     .......S........AAAV..L.....T.L.EA..XXXXDASNKFC.F............................................AHMNAQF  389
Daruma    ................AAAV..L.....T.L.QA.NXXXXDTSNKF..F............................................AHMNAQF  389
R54       ................AAAV..L.....T.L.EA.NXXXXDSSNKFV.F...........................................         327
GD19C     ................A.AVM.L.....T.L.EA.M.XXXDTSNKF..F..................L.I.......................AHMNAQF  389
C1-97     ................A.AVM.L.....T.L.EA.M.XXXDTSNKF..F..................L.I.......................AHMNAQF  389
CG1       ................A.AVM.L.....T.L.EA.I.XXXDTSNKF..F...............S                                  354
CW2       ................A.AVM.L.....T.L.EA.I.XXXDTSNKF..F...............S                                  354
HB1043    ................A.AVM.L.....T.L.EA.I.XXXDTSNKF..F..................L.I.......................AHMNAQF  389
CS1       ................A.AVM.L.....T.L.EA.I.XXXDTSNKF..F...............S                                  354
CP16      ................A.AVM.L.....T.L.EA.I.XXXDTSNKF..F...............S                                  354
CB7       ................A.AVM.L.....T.L.EA.I.XXXDTSNKF..F...............S                                  354
CE1       ................A.AVM.L.....T.L.EA.I.XXXDTSNKF..F...............S                                  354
CB8       ................A.AVM.L.....T.L.EA.I.XXXDTSNKF..F...............S                                  354
CB3       ................A.AVM.L.....T.L.EA.I.XXXDTSNKF..F...............S                                  354
```

+ Majority

Majority	- - -	
C11		350
MFR002IR		308
27		334
84-55	RF.	403
258		340
SP02	RF.	403
B4		374
A13		377
Cps2		374
KMZ04	RF.	403
Sku_Ps1	RF.	403
Raptor2-KU210		328
A12		375
C12		376
Raptor4-KU213		328
Raptor1-KU197		328
6BC	RF.	403
INEI-H-113-18		338
EAE_A22M	RF.	403
Cal-10	RF.	403
WS-RT-E30	RF.	403
06-859-1		346
2806-48	RF.	403
KKCP-1	RF.	403
Cps5		376
CP3	RF.	403
JS2020-5		342
GD19A1	RF.	403
SD_P5	RF.	403
Cps4		381
SZ15	RF.	403
CPS-QD-LS	RF.	403
GD19A3	RF.	403
JS2020-1		347
JS2020-2		343
LS-QXC01	RF.	403
19	RF.	403

GD19A2	RF.	403
SZ18-1	RF.	403
Mat116	RF.	403
M56	RF.	403
CPX0308	RF.	392
WC	RF.	397
92-1293		356
NJ1	RF.	391
TT3	RF.	393
6N		318
1V		290
7778B15		355
VS225	RF.	392
Daruma	RF.	392
R54		327
GD19C	RF.	392
C1-97	RF.	392
CG1		354
CW2		354
HB1043	RF.	392
CS1		354
CP16		354
CB7		354
CE1		354
CB8		354
CB3		354

Decoration 'Decoration #1': Hide (as '.') residues that match the Consensus exactly.
